# *DDX3X*-related neurodevelopmental disorder in males – presenting a new cohort of 19 males and a literature review

**DOI:** 10.1038/s41431-025-01832-x

**Published:** 2025-03-31

**Authors:** Milou G. P. Kennis, Dmitrijs Rots, Arjan Bouman, Charlotte W. Ockeloen, Caroline Boelen, Carlo L. M. Marcelis, Bert B. A. de Vries, Mariet W. Elting, Quinten Waisfisz, Mohnish Suri, Esperanza Font-Montgomery, Dawn S. Peck, Deirdre E. Donnelly, R. Curtis Rogers, Ruth Richardson, Roseline Caumes, Boris Chaumette, Cécile Louveau, Suzanne C. E. H. Sallevelt, Saskia M. Maas, Jeroen J. Smits, Mieke M. van Haelst, Rebecca J. Levy, Helen Stewart, Bart L. Loeys, Rolph Pfundt, Tjitske Kleefstra, Lot Snijders Blok

**Affiliations:** 1 Department of Human Genetics, Radboudumc, Nijmegen, The Netherlands; 2 Department of Clinical Genetics, Erasmus MC University Medical Center, Rotterdam, The Netherlands; 3 Children’s Clinical University Hospital, Riga, Latvia; 4 Department of Pediatrics, Admiraal De Ruyter Ziekenhuis, Goes, Zeeland The Netherlands; 5 Department of Human Genetics, Amsterdam UMC, Amsterdam, The Netherlands; 6 Department of Clinical Genetics, Nottingham University Hospitals NHS Trust, Nottingham, UK; 7 Department of Pediatrics/Genetics/Metabolism Disorders, Children’s Hospital of Michigan, Detroit, MI USA; 8 Department of Pediatrics, University of Missouri School of Medicine, Columbia, MO USA; 9 Northern Ireland Regional Genetics Centre, Belfast Health and Social Care Trust, Belfast, Northern Ireland; 10 Greenwood Genetic Center, Greenwood, SC USA; 11 Northern Genetics Service, Newcastle upon Tyne hospitals NHS Foundation Trust, Newcastle Upon Tyne, UK; 12 CHU Lille, Clinique de génétique Guy Fontaine, F-, 59000 Lille, France; 13 Université Paris Cité, Institute of Psychiatry and Neurosciences of Paris (INSERM U1266), GHU Paris Psychiatrie et Neurosciences, Paris, France; 14 Paediatric and Reproductive Genetics Unit, Women’s and Children’s Hospital, Adelaide, SA Australia; 15 Department of Human Genetics, University Medical Center Utrecht, Utrecht, The Netherlands; 16 Neurology and Neurological Sciences, Division of Child Neurology, Stanford University and Lucile Packard Children’s Hospital, Palo Alto, California, USA; 17 Oxford Centre for Genomic Medicine, Oxford University Hospitals NHS Foundation Trust, Oxford, UK; 18 Center of Medical Genetics, University of Antwerp and Antwerp University Hospital, Antwerp, Belgium; 19 Vincent van Gogh Institute for Psychiatry, Centre of Excellence for Neuropsychiatry, Venray, The Netherlands

**Keywords:** Rare variants, Genetic testing, Neurodevelopmental disorders

## Abstract

*DDX3X*-related neurodevelopmental disorder is one of the most common monogenic causes of intellectual disability in females, with currently >1000 females diagnosed worldwide. In contrast, reports on affected males with *DDX3X* variants are scarce. The limited knowledge on this X-linked disorder in males hinders the interpretation of hemizygous *DDX3X* variants in clinical practice. In this study, we present a new cohort of 19 affected males (from 17 unrelated families) with (possibly) disease-causing *DDX3X* variants, for whom we collected clinical and molecular data. Additionally, we reviewed the existing literature on 13 males with *DDX3X* variants. The phenotype in males is diverse, including intellectual disability, speech/language delays, behavioural challenges and structural brain abnormalities. The vast majority of males have missense variants, including two recurrent variants (p.(Arg351Gln) and p.(Arg488Cys)). No truncating variants have been reported, consistent with the presumed embryonic lethality of complete loss-of-function of DDX3X in males. In our novel cohort, 6/17 variants are de novo in the affected male and 3/17 variants are de novo in the mother. This study provides significant insights in the genetic and phenotypic spectrum of males with *DDX3X* variants, by presenting the data of a combined cohort (*n* = 32) of novel and published individuals. Our data show that variants in *DDX3X* can cause an X-linked neurodevelopmental disorder in males, with unaffected or mildly affected carrier females. These findings will aid the interpretation of hemizygous missense variants in *DDX3X* and can guide clinical management and counselling, in particular with regard to recurrence risks in the respective families.

## Introduction

*DDX3X* is an essential gene located on the X-chromosome (Xp11.4), that partially escapes X-inactivation [1, 2]. It encodes a DEAD-box RNA helicase with numerous important functions in cellular processes, including transcription regulation, splicing, RNA metabolism, translation, DNA repair, stress granule formation, inflammation and regulation of the Wnt/β-catenin pathway [3–6]. Research on *DDX3X* is extensive and spans across diverse disciplines and topics (including fundamental cell biology, oncology, immunology and neurodevelopmental disorders), indicating its broad functions and associations. *DDX3X*-related neurodevelopmental disorder (NDD) was first described in 2015 and has been identified as one of the most frequent monogenic causes of intellectual disability (ID) in females [7–11]. It is associated with a broad phenotypic spectrum in affected females, including ID with speech/language problems, behavioural issues, hypotonia, structural brain anomalies, epilepsy and movement disorders [8, 9, 12–17].

To date, several cohorts of females with *DDX3X*-related NDD have been described in literature [3, 7–9, 13, 14]. Multiple types of de novo variants have been identified in affected females, including nonsense, frameshift, missense and splice site variants. In contrast to the numerous reported females with *DDX3X*-related NDD, only a small number of reports on males with rare hemizygous *DDX3X* variants have been published [7, 18, 19]. All of these males have missense or splice site variants, either maternally inherited or de novo. So far, no germline truncating variants in *DDX3X* have been identified in males, indicating the presumed embryonic lethality of complete loss-of-function of DDX3X in the hemizygous state.

Currently, the limited knowledge on *DDX3X*-related NDD in males hampers the interpretation of rare hemizygous *DDX3X* variants. This is particularly unfavourable given the current increase in the discovery of variants of uncertain significance, due to the expanding use of next generation sequencing in patients with developmental disorders [20]. Furthermore, in two recent studies assessing the pathogenicity of *DDX3X* variants, no additional evidence was found for pathogenicity of a subset of *DDX3X* missense variants reported in males [21, 22].

With this study we aim to gain more knowledge on the genetic and phenotypic spectrum of *DDX3X*-related NDD in males. We here present the largest male cohort to date (*n* = 19) and a literature review of previously published individuals (*n* = 13). This comprehensive overview of 32 individuals will facilitate variant interpretation in males with rare hemizygous *DDX3X* variants and guide clinical management. Our study has important consequences for counselling of the respective families, in particular with regard to the recurrence risks of these X-linked variants.

## Materials and methods

### Study participants and data collection

The new cohort presented in this study consists of male individuals with ID/NDD and rare variants in *DDX3X* identified in a diagnostic setting (except for individual 10 - identified in a research setting) and reported as possibly contributing to the phenotype. Information on these individuals was assembled from clinicians through (inter)national collaborations, including the international and Dutch DDX3X foundations, between February 2022 and October 2024. De-identified clinical and variant data were collected from the involved clinicians through a standardized questionnaire. Informed consent for the sharing and publication of medical data was provided by patients or their legal representatives. Consent for publication of photographs was obtained separately. Genetic testing and research were performed in accordance with protocols approved by the local Institutional Review Boards.

### Literature review

A PubMed literature search was performed to identify published reports on male individuals with *DDX3X*-related neurodevelopmental disorder. Articles published until October 1st 2024 were identified using the following search strategy: ‘(DDX3X[Title/Abstract]) AND ((male* [Title/Abstract]) OR (boy [Title/Abstract]) OR (hemizygous [Title/Abstract]) OR (XY [Title/Abstract]))’. By screening the titles and abstracts, we selected relevant articles which included at least one male with a possibly disease-causing variant in *DDX3X* and included clinical information about the individual. We extracted genetic and phenotypic data on the male individuals from these papers and their supplementary data. For three males we received additional or updated information on clinical features after approaching the corresponding authors of the selected manuscripts (Supplementary Table S1).

### Next-generation sequencing and in silico variant analyses

The *DDX3X* variants in the probands in our cohort were identified by exome sequencing (individuals 1,3-14 and 17) or genome sequencing (individual 2, 15, 16) in a clinical setting. The variants were classified as pathogenic, likely pathogenic or variant of unknown significance (VUS) by the local genetic laboratory. The individuals did not have clear alternative genetic diagnoses explaining the NDD (except for individual 4, see Results). Inheritance of the variants was examined either as part of trio exome/genome sequencing or by Sanger sequencing of the specific variant in other family members. In this study, variants are annotated in *DDX3X* transcript NM_001356.5 (MANE transcript). In silico variant analyses to assess pathogenicity were performed using Alamut Visual Plus (Version 1.7.1), CADD (v1.7), PolyPhen-2, PhyloP, SIFT scores, MetaDome (Version 1.0.1) tolerance landscapes, AlphaMissense, SpliceAI [23] and DDGun (BioFold). Population allele frequencies of the variants were extracted from gnomAD (v4.1.0, accession date 16-10-2024). We reclassified all variants based on a combination of ACMG/AMP [24] and additional criteria (Supplementary Table S2).

### Three-dimensional protein structure analysis

The possible effects of the variants on DDX3X protein 3D structure were analysed using solved crystal structures of the (1) unbound DDX3X (bound to AMP) (PDB:5E7J [25]); (2) DDX3X dimer bound to dsRNA in pre-unwound state (PDB:6O5F [26]); and (3) DDX3X in post-unwound state (PDB:7LIU [not published] and 2DB3 [27]). All available structures lack the N-terminal part of the protein, so it was evaluated using the available AlphaFold2 structure [28]. The analysis was performed using the YASARA Structure v20 [29].

## Results

### Cohorts

**Fig. 1:**
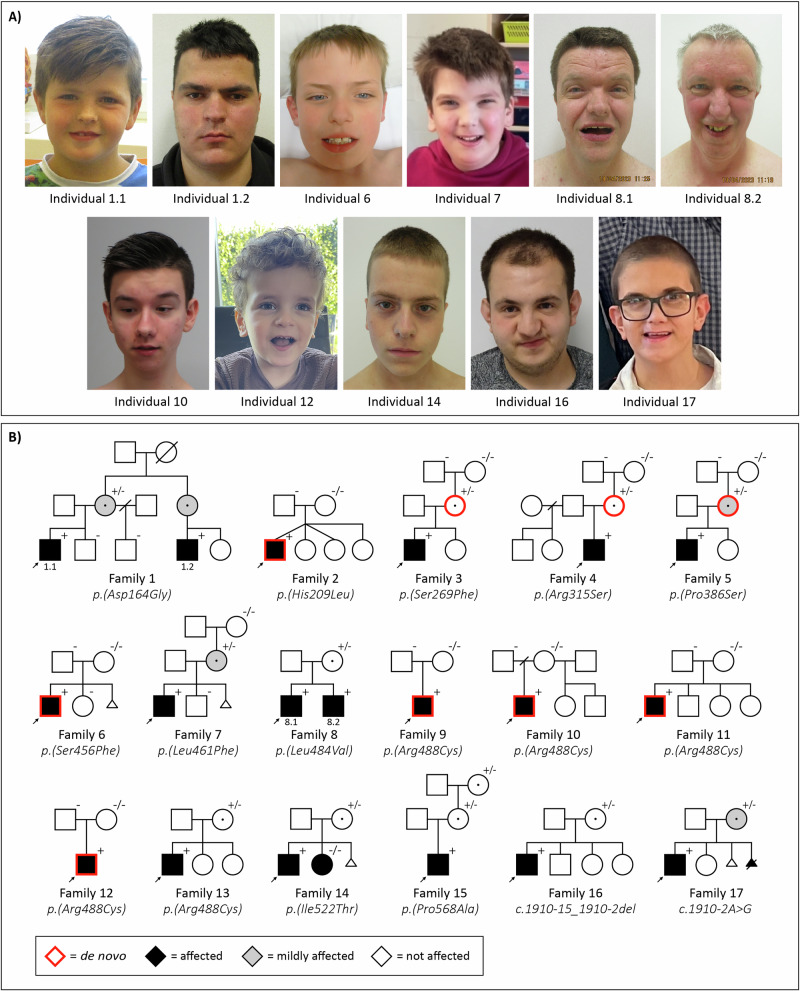
Facial photographs and pedigrees of males with *DDX3X* variants. **A** Facial photgraphs. **B** Pedigrees. All (relevant) tested family members are indicated by + (*DDX3X* variant present) or - (*DDX3X* variant absent) (noted as x/x in females for biallelic state).

**Fig. 2:**
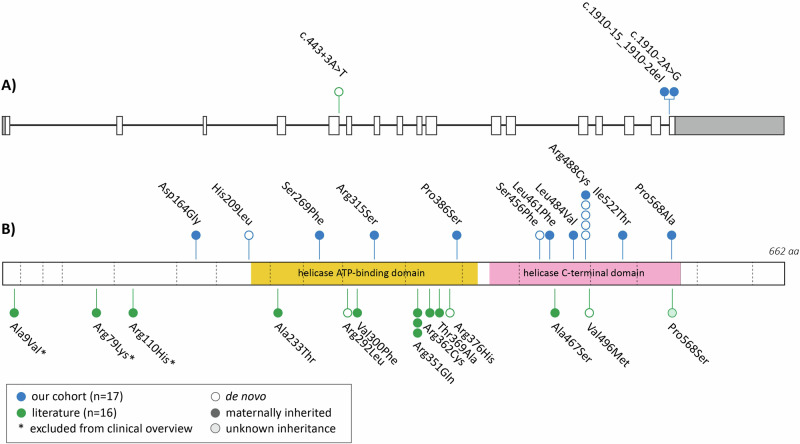
*DDX3X* variants in males. **A** Visual representation of *DDX3X* transcript (MANE transcript; NM_001356.5) with variants predicted to affect splicing. **B** Linear representation of the DDX3X protein including the two helicase domains (extracted from Uniprot) with locations of missense variants annotated.

In this study, we present a novel cohort consisting of 19 males (from 17 unrelated families) with rare hemizygous (possibly) disease-causing variants in *DDX3X*. We also reviewed the existing literature and found seven articles which in total described 16 male probands with *DDX3X* variants [3, 7–9, 14, 18, 19]. To enable a reliable overview of clinical features, we excluded three individuals from the previous literature carrying variants with low certainty of pathogenicity (p.(Ala9Val), p.(Arg79Lys) and p.(Arg110His)). In Table 1, we provide a consolidated clinical summary encompassing both cohorts (*n* = 32). Additionally, facial photos and pedigrees of males from our novel cohort are presented in Fig. 1. All variants identified in our novel cohort and previous literature are listed in Table 2 and Fig. 2. In the subsequent paragraphs, we will mainly focus on the males from our novel cohort.

**Table 1:** Clinical features in male individuals with *DDX3X* variants.

	Our cohort (*n* = 19)	Literature (*n* = 13) [3, 7–9, 14, 18, 19]	Combined (*n* = 32)
**Development**			
Developmental delay	19/19	9/9	28/28 (100%)
Intellectual disability	13/15	10/10	23/25 (92%)
Mild	6/13	1/10	7/23
Moderate	3/13	3/10	6/23
Moderate-severe	0/13	2/10	2/23
Severe	2/13	2/10	4/23
Unknown	2/13	2/10	4/23
Speech/language delays	19/19	5/5	24/24 (100%)
Motor development delays	17/18	3/3	20/21 (95%)
Behavioural problems	14/18	8/10	22/28 (79%)
ASD	6/18	2/4	8/22 (36%)
ADHD	3/16	2/3	5/19 (26%)
**Neurology**			
Hypotonia	8/16	7/7	15/23 (65%)
Hypertonia	4/12	0/3	4/15 (27%)
Movement problems	14/18	5/7	19/25 (76%)
Ataxia/coordination problems	6/18	NR	6/18 (33%)
Epilepsy	1/17	1/6	2/23 (9%)
Structural brain abnormalities	10/15	6/7	16/22 (73%)
Corpus callosum abnormalities	5/15	4/7	9/22 (41%)
Ventriculomegaly	4/15	1/7	5/22 (23%)
Microcephaly	0/19	4/9	4/28 (14%)
Macrocephaly	3/19	0/9	3/28 (11%)
**Other**			
Vision problems	11/19	5/9	16/28 (57%)
Refractive errors	8/19	2/9	10/28 (36%)
Strabismus	4/19	3/9	7/28 (25%)
Hearing/ear abnormalities	9/18	4/7	13/25 (52%)
Conductive hearing loss	3/18	2/7	5/25 (20%)
Recurrent ear infections	9/18	2/7	11/25 (44%)
Skeletal abnormalities	6/17	NR	6/17 (35%)
Joint hypermobility	4/17	0/2	4/19 (21%)
Cardiac abnormalities	4/17	4/9	8/26 (31%)
Respiratory abnormalities	5/17	0/1	5/18 (28%)
Gastrointestinal problems	10/17	3/5	13/22 (59%)
Urogenital abnormalities	6/18	3/4	9/22 (41%)
Cryptorchidism	3/18	1/4	4/22 (18%)

*NR* Not reported.

**Table 2:** In silico prediction tools and classification of *DDX3X* variants in males.

	cDNA [NM_001356.5]	Protein effect	Inheritance	CADD PHRED	Poly Phen2	SIFT	DDG	AlphaMissense [31]	Splice AI	gnomAD v4.1.0	ClinVar*	Class**
**This study**												
Individual 1.1 / 1.2	c.491 A > G	p.(Asp164Gly)	mat	27.7	D	D	N	D	N	No	No	3
Individual 2	c.626 A > T	p.(His209Leu)	dn	25.3	PD	D	N	D	N	No	No	4
Individual 3	c.806 C > T	p.(Ser269Phe)	mat (m dn)	31	D	D	N	D	N	No	No	4
Individual 4	c.943 C > A	p.(Arg315Ser)	mat (m dn)	28.1	D	D	N	D	N	No	No	3
Individual 5	c.1156 C > T	p.(Pro386Ser)	mat (m dn)	23.9	D	D	N	D	N	No	No	4
Individual 6	c.1367 C > T	p.(Ser456Phe)	dn	24.8	PD	D	N	N	N	No	No	4
Individual 7	c.1383 A > T	p.(Leu461Phe)	mat	14.57	D	D	D	D	N	No	No	3
Individual 8.1 / 8.2	c.1450 C > G	p.(Leu484Val)	mat	26.2	D	D	N	D	N	No	No	3
Individuals 9-13	c.1462 C > T	p.(Arg488Cys)	4 dn / 1 mat	31	D	D	D	D	N	No	Yes; het + hemi	5
Individual 14	c.1565 T > C	p.(Ile522Thr)	mat	25.8	PD	D	D	D	N	Yes; 1 het	No	3
Individual 15	c.1702 C > G	p.(Pro568Ala)	mat (m + )	26.5	D	D	N	D	N	No	No	3
Individual 16	c.1910-15_1910-2del	p.?	mat	x	x	x	x	x	D	No	No	3
Individual 17	c.1910-2 A > G	p.?	mat	x	x	x	x	x	D	No	No	3
**Previous literature**												
Parra (2024) [43] ***	c.26 C > T	p.(Ala9Val)	mat	20.4	N	N	x	N	N	No	NA	3
Kellaris (2018) [19] ***	c.236 G > A	p.(Arg79Lys)	mat	24.2	N	N	x	N	N	Yes; 8 het, 2 hemi	NA	3
Dai (2022) [14] ***	c.329 G > A	p.(Arg110His)	mat	24.2	D	D	x	N	N	No	NA	3
Wang (2018) [8]	c.443+3 A > T	p.?	dn	x	x	x	x	x	D	No	NA	3
Parra (2024) [43]	c.697 G > A	p.(Ala233Thr)	mat	31	D	D	N	D	PD	No	NA	3
Tang (2021) [9]	c.875 G > T	p.(Arg292Leu)	dn	34	D	D	N	D	N	No	NA	4
Snijders Blok (2015) [7]	c.898 G > T	p.(Val300Phe)	mat (m dn)	31	D	D	N	D	N	No	NA	3
Snijders Blok (2015) [7], Wang (2018) [8], Lennox (2020) [3]	c.1052 G > A	p.(Arg351Gln)	2 mat / 1 unk	25.5	PD	D	N	D	N	No	NA	4
Snijders Blok (2015) [7]	c.1084 C > T	p.(Arg362Cys)	mat	24.8	D	D	N	D	N	No	NA	3
Lennox (2020) [3]	c.1105 A > G	p.(Thr369Ala)	unk	21.3	N	D	N	N	N	No	NA	3
Nicola (2019) [18]	c.1127 G > A	p.(Arg376His)	dn	24.9	D	D	N	D	N	No	NA	4
Lennox (2020) [3]	c.1399 G > T	p.(Ala467Ser)	mat (m + )	23.3	N	D	N	N	N	No	NA	3
Nicola (2019) [18]	c.1486 G > A	p.(Val496Met)	dn	28.4	D	D	D	D	N	No	NA	4
Nicola (2019) [18]	c.1702 C > T	p.(Pro568Ser)	unk	26.5	D	D	N	D	N	No	NA	3

Inheritance: *mat* maternally inherited, *dn* de novo, *(m dn)* de novo in carrier mother, *(m* *+* *)* grandmother carrier, *unk* unknown. Scores prediction tools: *D* (Likely) Damaging, *N* Normal / Not damaging, *PD* Possibly damaging (see Table S2). Other abbreviations: *AL* Acceptor Loss. *CADD* Combined Annotation Dependent Depletion-Phred (v1.7), *DDG* Delta Delta G, *hemi* hemizygous, *het* heterozygous, *SIFT* Sorting Intolerant From Tolerant. *Only applicable for the new cohort. **See Supplementary Table S2 for details. ***Not included in clinical data (doubts about pathogenicity).

### Clinical characteristics

The 19 male individuals in our new cohort show overlapping neurodevelopmental symptoms with variable other features, summarized in Table 1 (details in Supplementary Table S1). The median age at detection of the *DDX3X* variant was 8 years (range 9 months – 47 years). In 12/17 individuals, abnormalities were present during pregnancy and/or delivery. This includes abnormalities on prenatal ultrasounds (4/17), (partial) placental abruption (3/17) and a caesarean section for various reasons (7/17). All males have developmental delays and/or an intellectual disability, which is mild in six, moderate in three and severe in two individuals (and of unknown severity in eight). IQ data are available for five individuals, demonstrating a mean total IQ of 65 (range 56–75). All males have a delayed speech and language development; the age of first words ranges from 18 months to 8 years (median 24 months). Three males have verbal dyspraxia or speech apraxia. Regarding current speech and language capacities, 4/7 individuals (aged 4 years and older) are minimally verbal, with most only speaking a few words. Motor development is delayed in 17/18 individuals, with a median age of first steps at 24 months. Behavioural difficulties are present in 14/18 individuals. Six individuals have officially been diagnosed with an autism spectrum disorder (ASD); additionally six males show features of ASD without a formal diagnosis. Three individuals have a formal diagnosis of ADHD, and two additional males show signs of ADHD.

Regarding neurological features, both hypotonia and hypertonia are reported (8/16 and 4/12 respectively). About three quarters of the individuals (14/18) experience movement problems: six show ataxia or coordination problems. Also, abnormal gait, stiff legs and frequent falls are described repeatedly. One male (individual 7) had epilepsy in the past (complex partial epilepsy), for which he received anti-epileptic medication. Three others experienced a single (febrile) seizure and one had staring spells. A cerebral MRI or CT scan shows abnormalities in ten (out of 15) males, including corpus callosum abnormalities (5/15), enlarged ventricles or hydrocephalus (4/15) and a small or thin brainstem (2/15). One individual has a retrocerebellar arachnoidal cyst, another has a Blake’s pouch cyst.

Vision problems (11/19) consist mainly of mild refractive errors, strabismus and amblyopia. Nine individuals have recurrent ear infections and/or a need for ear tubes. Four individuals have diverse congenital cardiac anomalies, encompassing a ventricular septal defect, a combination of a ventricular septal defect and atrial septal defect, a bicuspid aorta valve and an aberrant right subclavian artery. Gastrointestinal problems include gastroesophageal reflux (4/17), faecal incontinence (3/17) and constipation (3/17). Six males have one or more urogenital abnormalities, including cryptorchidism (3/18), inguinal hernia (2/18) and a hydrocele (3/18). Plagiocephaly is present in three individuals, and one male has a cleft lip-palate. One individual has a humerus cyst; another has an osteochondral defect of the left talus. No malignant neoplasms have been reported. Variable dysmorphic facial features are present in the cohort, but we did not observe a clear recognisable facial phenotype (Fig. 2A). However, a prominent/high/broad forehead, deep-set eyes and prominent ears are reported in multiple individuals.

Interestingly, five out of the twelve carrier mothers in our cohort report mild neurodevelopmental symptoms, which are notably less severe than in their affected sons. Four mothers have mild learning problems, requiring special education in two; one also has ADHD; and one has dyslexia. Two carrier mothers report a single early miscarriage in the past, whilst the mother of individual 16 had multiple early miscarriages. Also, she underwent a termination of pregnancy involving a male foetus who had cardiovascular abnormalities and Dandy-Walker syndrome; no genetic test was performed at the time.

In two families, an additional affected male was identified and confirmed to carry the *DDX3X* variant. This involves a cousin of the index (in family 1) and a brother (in family 8), whose clinical features are also part of this study. In family 14, the affected male has a sister with intellectual disability, autism spectrum disorder, coarse facies and a small stature. Genetic testing showed that she does not carry the *DDX3X* variant, and whole exome sequencing was negative.

### Variants and in silico analyses

In the 17 probands in our novel cohort, thirteen unique variants were observed (Fig. 2 and Table 2). Eleven variants lead to amino acid substitutions (missense variants), while two affect a canonical splice site of the last exon. Intriguingly, one of the missense variants (p.(Arg488Cys)) is present in five unrelated individuals. Three missense variants lead to amino acid substitutions located in the helicase ATP-binding domain, six in the helicase C-terminal domain and two variants are located outside of the known functional domains of the DDX3X protein (one of which is within the Q motif just prior to the helicase ATP-binding domain).

For all variants in our new cohort, segregation analysis (or trio WES/WGS) was performed to investigate the inheritance of the variants (Fig. 1B). Six variants were found to be de novo in the affected male (four of which concerned the recurrent p.(Arg488Cys) variant), and 11 variants were found to be maternally inherited. In five of the families with maternally inherited variants, further segregation analysis was performed in the maternal grandparents. In three of these families, the *DDX3X* variant was found to be de novo in the mother of the proband (family 3-5); in one family the grandmother did not have the variant but the grandfather was unavailable for testing (family 7); and in one family the variant was inherited from the maternal grandmother (family 15). In family 1, the maternal grandmother was not tested, but the variant was most likely inherited from her because of the occurrence in two male cousins and their mothers, although germline mosaicism in the grandmother cannot be excluded. X-inactivation tests were performed in four carrier mothers, and showed random X-inactivation in two mothers (family 4 and 14) and extreme skewing in two (family 8 and 16).

A diverse range of in silico prediction tools was used to aid variant interpretation for all variants in our cohort and previously reported males (Table 2 and Supplementary Table S2). All variants from our novel cohort had a CADD PHRED (v1.7) score ≥23, besides the p.(Leu461Phe) with a CADD score of 15.08 (other prediction scores pointed to pathogenicity for this variant). All variants were absent from gnomAD (v4.1.0), except for p.(Ile522Thr) which was present once in a heterozygous state. The two splice site variants (c.1910-15_1910-2del and c.1910-2 A > G) are both predicted to cause loss of the canonical acceptor site of the last exon. According to prediction tools, a new acceptor site (canonical in alternative transcript ENST00000625837.2) might get activated. However, the exact effects of these variants on transcript and protein levels are currently unknown. In individual 4 another genetic variant possibly contributing to the phenotype was found: a likely pathogenic variant in *KMT2C* (Supplementary Table S1). Since the phenotypes of *DDX3X*-related NDD and *KMT2C*-related NDD are rather aspecific and overlapping, it is difficult to determine the relative contributions of both variants to this individual’s phenotype [30].

In the 16 male probands with *DDX3X* variants from previous literature (of which we excluded three for the clinical overview), 14 unique variants were reported, including 13 missense and one splice site variant (Fig. 2). In five individuals, the variant was confirmed to be de novo. The p.(Arg351Gln) variant was recurrently identified in three unrelated males. Similar to our new cohort, we found a clustering of missense variants within the two helicase domains. Interestingly, the p.(Arg568Ala) affects the same amino acid position as the variant found in individual 15 within our cohort.

We systematically reclassified all *DDX3X* variants from our novel cohort and previous literature, ranging from class 3 (variant of unknown significance) to class 5 (pathogenic) (Table 2 and Supplementary Table S2).

### Three-dimensional protein structure analysis

DDX3X is a dynamic protein with multiple conformations and binding partners. Therefore, the effects of the variants were predicted on several available DDX3X conformational structures (see Materials and Methods). Variant analysis showed that the identified missense variants likely exhibit different molecular mechanisms of (partial) loss of the functions of DDX3X.

Out of 24 analysed missense variants, eight variants were predicted to disrupt and/or destabilize one of the helicase domain’s structure: p.(His209Leu), p.(Ala233Thr), p.(Ser269Phe), p.(Val300Phe), p.(Leu461Phe), p.(Val496Met), p.(Pro568Ala) and p.(Pro568Ser) (see Supplementary Fig. S1 and Supplementary Table S3). In addition, four variants were predicted to affect the protein’s conformation changes and/or stability in a specific conformational state: p.(Asp164Gly), p.(Pro386Ser), p.(Leu484Val) and p.(Arg488Cys). Nine of the identified missense variants were located at or near the surface of the DDX3X protein, with four variants predicted to affect binding to the second DDX3X monomer in a homodimer or binding to the RNA: p.(Arg292Leu), p.(Arg351Gln), p.(Ser456Phe), p.(Ile522Thr). The five remaining variants located at or near the surface (p.(Arg315Ser), p.(Arg362Cys), p.(Thr369Ala), p.(Arg376His) and p.(Ala467Ser)) were not predicted to affect surfaces that are known to bind to other proteins or RNA, so their effect on the protein is currently unknown. Similarly, the N-terminal part of the DDX3X contains a disordered region, so the three variants located in this region (p.(Ala9Val), p.(Arg79Lys), p.(Arg110His)) are not expected to affect the protein’s secondary structure. However, DDX3X is known to have dozens of binding partners for which exact binding sites are largely unknown. Therefore, we cannot exclude that the surface variants, as well as the N-terminal variants could disrupt binding to other proteins.

## Discussion

In this study, we provide the first overview of *DDX3X-*related neurodevelopmental disorder (NDD) in males, by presenting a large new cohort of affected males (*n* = 19) and a review of published patients in the literature (*n* = 13). We demonstrate the clinical and molecular features of this X-linked disorder in males.

The clinical spectrum in the males within our study is diverse. All males have a developmental delay and/or intellectual disability, ranging from mild to severe. Behavioural challenges, movement problems and brain abnormalities are frequent. Although the phenotype is rather non-specific and variable, clinical features are comparable to those described in females with *DDX3X*-related NDD [12]. Interestingly, 5/12 of the carrier mothers of affected males in our novel cohort have mild neurodevelopmental symptoms. In previous literature, most carrier mothers were thought to be asymptomatic.

Regarding the molecular spectrum of *DDX3X* variants, the vast majority of males (27/30) have missense variants and three males have splice site variants. No protein truncating variants have been reported, in contrast to affected females where these loss-of-function variants are very common [4]. In males, most missense variants are located within the helicase ATP-binding domain and helicase C-terminal domain. Two missense variants are recurrently present in males: the p.(Arg351Gln) is present in three unrelated males from the literature, and the p.(Arg488Cys) variant in five unrelated males in our novel cohort. Notably, the latter variant has been described in affected females too [31], and this amino acid position has been found to be recurrently mutated [3]. To our knowledge, this is the only *DDX3X* variant which has been reported in both male and female probands with NDD. Additionally, three variants predicted to affect splicing are present in our study; their precise effects on the protein are currently unknown. Ten out of the 27 unique variants presented in this study have also been reported as somatic variants in various forms of cancer [32]. No malignant neoplasms have been reported in the males in our study.

In our study, we see several indicators for pathogenicity of the *DDX3X* variants in males. Firstly, certain missense variants are recurrently present in affected males, whilst absent from population databases. Furthermore, the *DDX3X* variant was de novo in 10/30 males. Additionally, three of the maternally inherited missense variants in our novel cohort were confirmed de novo in the carrier mother.

Evidently, there are different patterns of variant types present between males and females with this X-linked disorder that partially escapes X-inactivation. In females, loss-of-function is an acknowledged mode of pathogenicity. In addition, a subset of recurrent missense variants demonstrate a dominant-negative effect causing aberrant RNP granules (generally associated with a more severe phenotype) [3]. In the hemizygous state, complete loss-of-function of DDX3X is thought to be embryonically lethal, consistent with results from animal studies [33, 34]. *DDX3Y*, a Y-chromosome homologue of *DDX3X* mainly expressed in the testis, seems unable to compensate for the loss of DDX3X [4]. We propose that the missense variants reported in affected males have a partial loss-of-function (or hypomorphic) effect, supported by previous functional studies in zebrafish [7, 19]. Assumably these variants cause a phenotype in the hemizygous state, whereas the effect on females will be milder due to the existence of a second unaffected allele and/or partial X-inactivation. Accordingly, carrier mothers are either asymptomatic or mildly symptomatic.

Several other X-linked conditions show similar differences in variant types between affected males and females, including disorders resulting from pathogenic variants in *USP9X*, *ALG13*, *KDM5C*, *IQSEC2*, *OFD1* and *SMC1A* [35–40]. These genes all (partially) escape X-inactivation, feature de novo loss-of-function variants in affected females, and milder (often maternally inherited) missense variants in males. We suggest to refer to this group of disorders (including *DDX3X*-related NDD) as ‘X-linked’, instead of X-linked dominant or X-linked recessive [41, 42].

In contrast to our findings, two recent studies did not find supporting evidence for pathogenicity of *DDX3X* variants in males. One study, using statistical enrichment analyses to identify X-linked NDD genes, showed a female bias for de novo mutations in *DDX3X* [21]. It is clear that the prevalence of de novo *DDX3X* variants is higher in females than in males, but in our view an absence of enrichment does not exclude the possibility of pathogenicity of rare variants in males. Another study, assessing pathogenicity of *DDX3X* variants using saturation genome editing (SGE) analyses, did not find supporting functional evidence for pathogenicity of most variants in males [22]. They suggest further evidence is needed to support the association between damaging *DDX3X* variants and NDD in males. We theorize that the effects of *DDX3X* missense variants in males might not have been picked up by these analyses, for example because the assay may not have been sensitive enough to identify variants with hypomorphic effects. In contrast to these two studies, our study provides substantial evidence supporting the occurrence of *DDX3X*-related NDD in males. While we cannot classify all variants as pathogenic at the moment, our data confirm that this disorder can affect males. For future research, we recommend additional functional studies for *DDX3X* variants to help further understanding the different modes of pathogenicity in both males and females.

In conclusion, this study shows that specific *DDX3X* variants in males can cause an X-linked neurodevelopmental disorder. Our overview and insights will aid in the interpretation of hemizygous *DDX3X* variants in clinical practice, and guide counselling and management in the corresponding males and their families.

## Supplementary information

Table S1 - Clinical characteristics

Table S2 - Variant characteristics

Table S3 - Three-dimensional protein structure analysis

Figure S1 - Three-dimensional protein structure analysis

## Data Availability

The datasets generated and used in this study are available in Supplementary Tables S1–S3. Additional information is available from the corresponding authors on reasonable request.
