# Supplementary figures and images for: DDX3X-related neurodevelopmental disorder in males – presenting a new cohort of 19 males and a literature review
Source: Eur J Hum Genet. 2025 Mar 31;33(8):980–8. doi: 10.1038/s41431-025-01832-x (PMC12322063; doi:10.1038/s41431-025-01832-x)

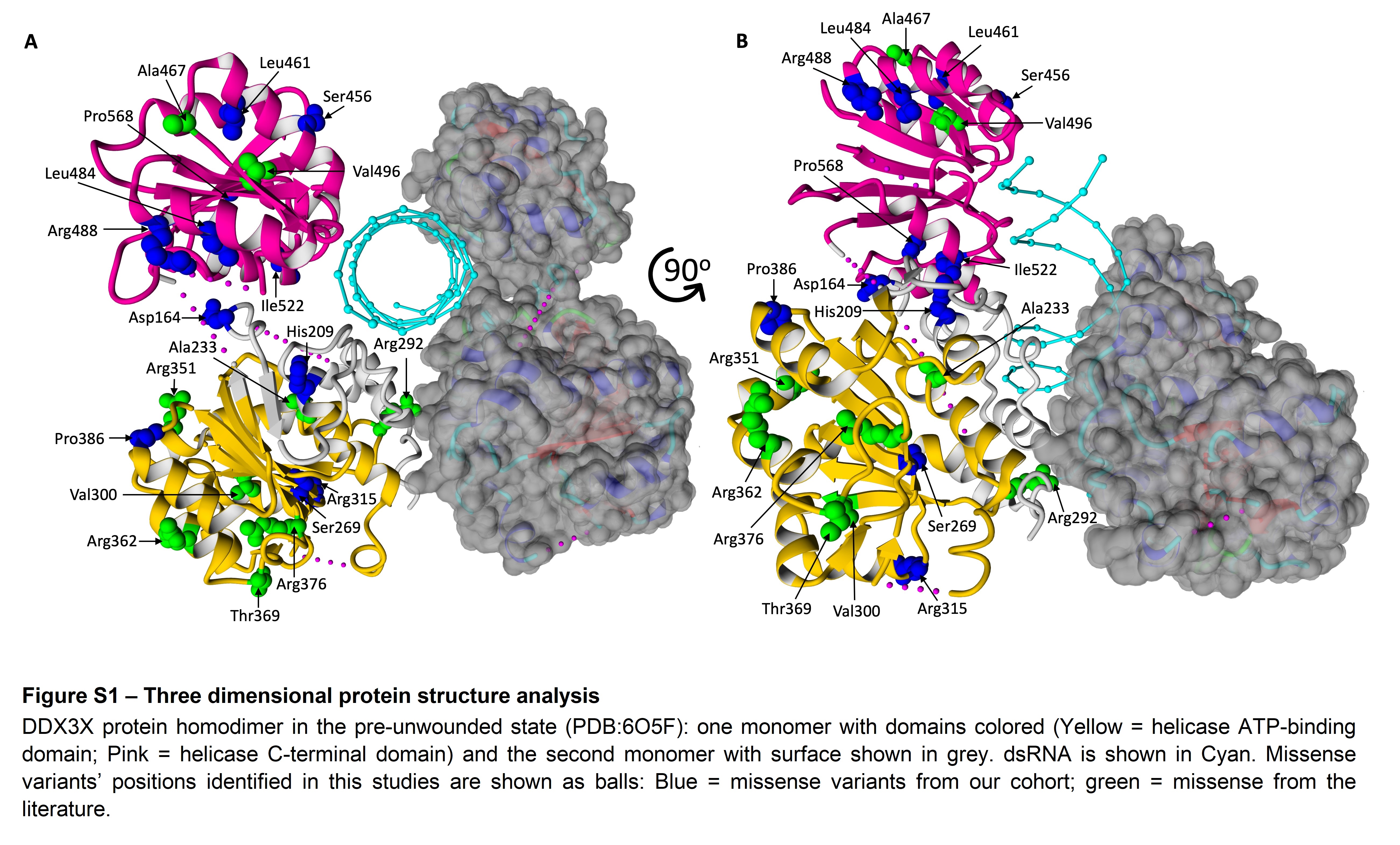

Supplement: Supplementary file 4 — Figure S1 - Three-dimensional protein structure analysis [file 41431_2025_1832_MOESM4_ESM.jpg]
